# Intercomparing varied erosion, deposition and transport process representations for simulating sediment yield

**DOI:** 10.1038/s41598-019-48405-9

**Published:** 2019-08-19

**Authors:** Tan Zi, Mukesh Kumar, John Albertson

**Affiliations:** 1 Tetra Tech Inc, Integrated Water Management, Fairfax, 22030 USA; 2 University of Alabama, Civil, Construction, and Environmental Engineering, Tuscaloosa, 35487 USA; 3 Cornell University, Civil and Environmental Engineering, Ithaca, 14853 USA

**Keywords:** Hydrology, Natural hazards

## Abstract

Over the past several decades there has been an enormous proliferation of sediment models, ranging from empirical to physically-based. Most of these models aim to capture the impacts of three primary sediment processes viz. erosion, deposition, and transport. As a range of process representations exist for simulating these three processes, it is natural to wonder about their influence on estimates of suspended sediment yield from a watershed. While several studies have focused on intercomparison of sediment models, their scopes have generally been restricted to comparing the individual model performances, rather than understanding the role of process representations on sediment model output. Here, six model configurations, which span the different permutations of erosion, deposition and transport process representations being used in extant models, are considered to evaluate the role of process representations on sediment yield estimates. The numerical experiments are designed to evaluate the extent to which the sediment dynamics as modeled by a physically-based model with coupled surface-subsurface hydrologic interactions are captured by simpler models. The presented work delineates the applicability and limitations of widely used representations of sediment processes, and could help users identify the pros and cons of using a sediment model at a given temporal scale.

## Introduction

Both empirical and physically-based models are widely used to estimate the erosion and sediment transport processes^1–4^. Empirical models generally provide erosion estimates at coarse temporal resolutions, but have the advantage of using fewer input parameters and being computationally efficient^5^. These models often assume that the properties of the watershed are stationary. In contrast, physically-based erosion models often provide estimates at fine temporal resolutions and can account for temporal variations in watershed properties and antecedent states. These models have more detailed descriptions of erosion and sediment transport processes^6–12^, and can account for the spatial heterogeneity of geomorphology, topography, soil and land surface properties, and the temporal variations in meteorological inputs. However, physically-based models are data and computation intensive. Data acquisition and uncertainty estimation costs associated with these models are also relatively large^13^. Many other pros and cons of empirical, semi-empirical and physically-based models vis-a-vis their input–output data requirements, model structure, uncertainty, and accuracy have been discussed in detail in several review studies^3,14–17^.

Irrespective of the model formulation, a majority of sediment models strive to capture the impacts of three primary processes viz. detachment or erosion, deposition, and transport. These processes are generally represented in markedly different forms in empirical, semi-empirical and physically-based models (Table 1).

**Table 1:** A broad classification of detachment, deposition and transport processes in sediment models.

Process Representations		
Detachment	Deposition	Transport
Type 1: Driven by rain erosivity (e.g. *EI30*) Type 2: Driven byflow erosivity (e.g. *f(Q, q*_*peak*_)) Type 3: Explicit evaluation of rainfall and flow detachment based on rainfall kinetic energy and flow shear stress.	Type A. No explicit evaluation of deposition Type B. Limited by SDR Type C. Limited by TC –Type C1. TC calculation based on rainfall erosivity and topographic attributes. –Type C2. TC calculation based onflow erosivity and topographic attributes. –Type C3. TC calculation based on explicit calculation of flow property and shear stress.	Type i. No routing calculation Type ii. Routing based on mass balance

For example, in empirical models, the potential soil erosion, which may be caused by raindrop impact and/or from overland flow shear on the soil surface, is quantified based on rainfall erosivity^18^. Rainfall erosivity (*R*_*e*_) is evaluated using:

1 $${R}_{e}\propto EI30$$

where *E* is rainfall energy and *I30* is maximum 30 minutes rainfall intensity. *R*_*e*_ implicitly captures the enhanced detachment during high intensity storms, both due to raindrop impact and shear stress from overland flow. For quick reference, explanation of relevant symbols used in the paper are also presented in the SI section titled “Symbols List”. Since antecedent conditions may influence the generation of overland flow volume and peak^19^, instead of only using derived precipitation statistics, several models^20^ directly use flow volume (*Q*) and peak flow rate (*q*_*peak*_) for flow erosivity (*F*_*e*_) calculations:

2 $${F}_{e}\propto f(Q,{q}_{peak})$$

Overland flow data used for erosivity calculations in these models are often generated using Soil Conservation Service (SCS) curve number method^21^. Physically-based models generally explicitly evaluate erosion due to both rain drop (*D*_*r*_) and flow detachment (*D*_*f*_) processes using:

3 $${D}_{E}={D}_{r}+{D}_{f}\propto f(E,(TC-C))$$

where f() is a functional relation, *D*_*E*_ is the total detachment, and *TC* and *C* are transport capacity and sediment concentration of flow. *D*_*r*_ is a function of rainfall energy (*E*), while *D*_*f*_  is a function of the gradient between *TC* and *C*. Assuming all other conditions to be the same, *D*_*f*_  decreases as this gradient reduces. Here *TC* is defined as the maximum concentration of sediment that can be carried with the flow. The gradient function used to calculate *D*_*f*_ is dependent on both flow characteristics such as velocity, volume and friction slope, and sediment grain properties such as its size and specific gravity^22,23^. Flow properties used for estimation of *D*_*f*_  in these models are sometimes obtained using coupled interactions between surface, subsurface and ecological processes^10,12^ and at other times based on simpler infiltration and runoff generation mechanisms such as using SCS curve number method or Green-Ampt method as done in WEPP and SWAT models^24,25^.

As far as deposition and transport processes are concerned, many empirical erosion and sediment yield models, e.g. Universal Soil Loss Equation (USLE)^18^ and Revised Universal Soil Loss Equation (RUSLE)^26^, were not designed to explicitly simulate these processes at all. However, RUSLE equation is used within several empirical models for sediment yield simulations. This generally involves use of a linear multiplier called sediment delivery ratio (SDR) to indirectly account for deposition during the sediment routing processes^27^. SDR approximates the average portion of eroded soil that is transported out of the area of interest. In many other models^28,29^, erosion estimated by USLE equation are routed downhill based on the transport capacity calculations. If transport capacity is smaller than the total amount of sediments, the “excess” sediment is deposited or else it is carried downstream. These models generally use long term (often annual) rainfall and peak flow statistics and topographic attributes to obtain transport capacity^30,31^. In physically-based models, the transport capacity of flow is calculated based on the evolving hydrological state at a given location^22,32^, and is a function of flow velocity and volume, and friction slope.

The representation of erosion, deposition and transport processes in empirical, semi-empirical and physically-based models are not restricted to the example configurations discussed above. Varied permutations of the process representations are often used in sediment models (see more details in Supplementary Table S2). Also, the temporal resolution at which these models are applied varies a lot. Given the range of process representations and multiple temporal scales at which they are applied for simulation of erosion, deposition, and transport processes, it is natural to wonder about the influence of simplifications in process representations on estimates of sediment yield. While several studies have performed intercomparison of sediment models^33–36^, they have mostly focused on comparing the performances of the model with respect to the observed data. Since the models used in comparison studies often have multiple differences between them, such as in the use of calibration strategy, domain discretization, parameters, data inputs, and above all in the representations of individual hydrologic and sediment processes, these studies were not well suited for understanding the role of different process representations on sediment model output. Here, an intercomparison experiment is designed to evaluate the role of commonly used permutations of representation of erosion, deposition and transport processes on modeled sediment yield estimates. The experiment allows assessment of the extent to which sediment dynamics as simulated by a physically-based model with coupled surface-subsurface flow interactions, are captured by models with simpler process representations. The following section presents the analyses and discussion of our results. Readers are suggested to first go over the Methods section at the end of this paper to get acquainted with the name abbreviations and details of the model configurations.

## Results and Discussion

### Intercomparing SSY estimates at multiple scales

**Figure 1:**
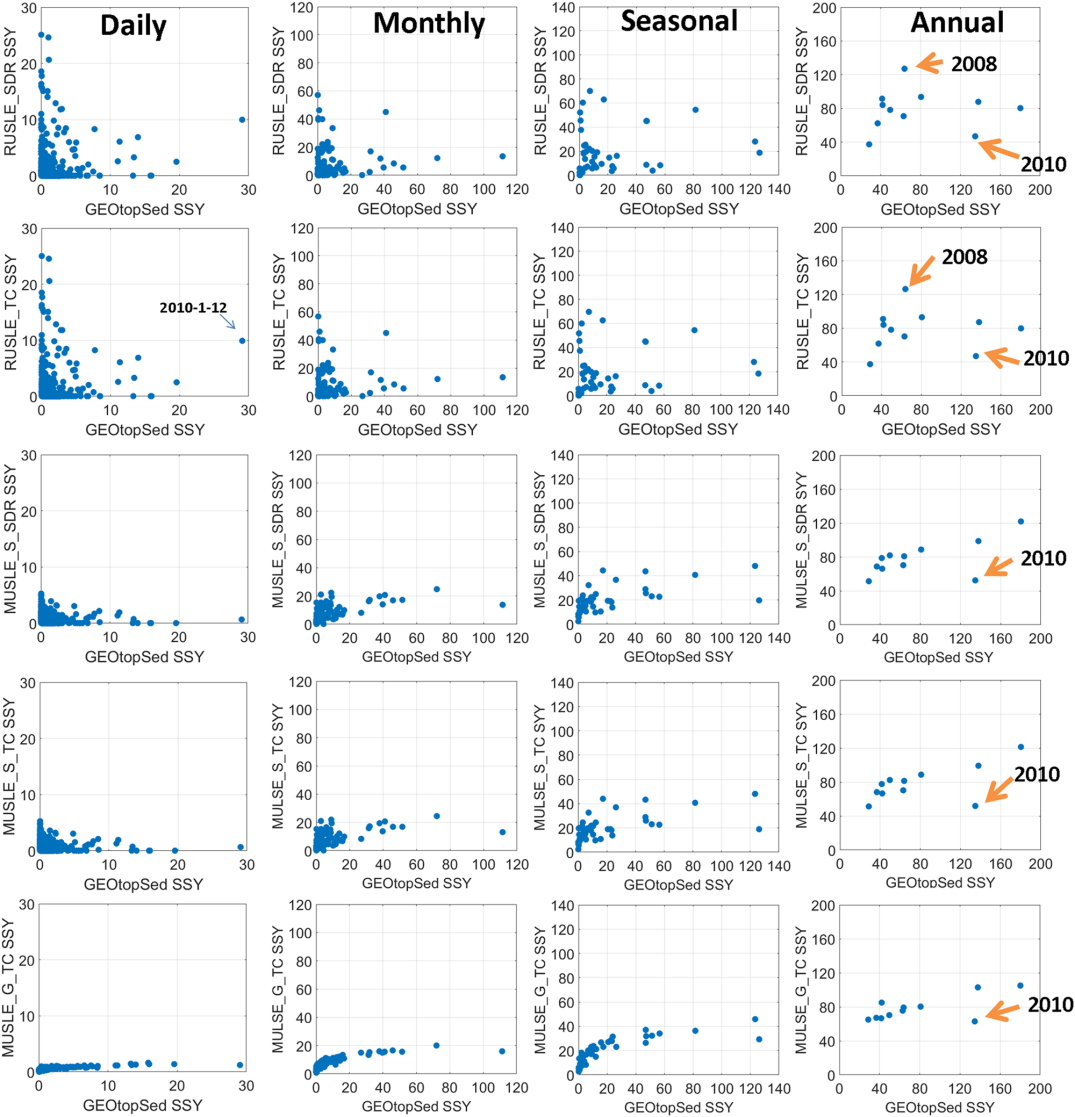
Scatter plots comparing SSY estimates (ton/time interval) between GEOtopSed and five other model configurations at daily, monthly, seasonal, and yearly scales. A zoom-in of individual panels is shown in Supplementary Fig. S2.

At the annual scale (Fig. 1), suspended sediment yield (SSY) estimates from the other five model configurations appear to closely co-vary with the SSY estimates obtained from GEOtopSed, except for a few years (e.g. 2008, 2010). If the year 2010, for which all model representations grossly underestimate the SSY with respect to GEOtopSed (Fig. 1), is not considered, the Fisher estimator between the GEOtopSed model and the other three MUSLE based model representations are all larger than 0.9 at annual scale (Table 2).

**Table 2:** Fisher unbiased estimator for simulated SSY at different temporal scales.

Model Comparisons	Annual	Seasonal	Monthly	Daily
MUSLE_G_TC vs GEOtopSed	0.67 (0.93)	0.77 (0.84)	0.74 (0.81)	0.63 (0.68)
MUSLE_S_TC vs GEOtopSed	0.64 (0.93)	0.58 (0.70)	0.56 (0.65)	0.26 (0.28)
RUSLE_TC vs GEOtopSed	0.04 (0.25)	0.15 (0.16)	0.15 (0.15)	0.13 (0.14)
MUSLE_S_SDR vs GEOtopSed	0.64 (0.93)	0.59 (0.70)	0.57 (0.65)	0.26 (0.29)
RUSLE_SDR vs GEOtopSed	0.04 (0.25)	0.15 (0.16)	0.15 (0.15)	0.13 (0.14)

Values in the parenthesis are Fisher estimator after discarding data from 2010.

This implies that for most years in Dripsey catchment, the annual estimates of SSY obtained using models based on MUSLE based schemes have high correlations (F > 0.6) with the estimates from a computationally intensive physically-based model, as long as the simulated hydrology in either model configuration is identical. The reasons for bias in the model performance in 2010, which received more than 112 mm rainfall within 10 days in January, is presented in the following section. The RUSLE based models (both RUSLE_TC and RUSLE_SDR), however, had F smaller than 0.25, thus implying that erosion estimates based on derived statistics of rainfall may produce inter-annual variations that are significantly different than those obtained by MUSLE based models or the physically-based model. At finer temporal scales (daily to seasonal), SSY estimates from MUSLE_G_TC model showed a power-law relation with the SSY estimates from the GEOtopSed model. For other four model configurations, the Fisher estimator reduced significantly from annual to intra-annual scales (Table 2). The correlation between GEOtopSed and other MUSLE based model representations is larger than 0.6 at seasonal and monthly scales, as long as the year 2010 is excluded. At daily scale, the correlation for all model representations (except MUSLE_G_TC) is less than 0.26 with 2010 data and less than 0.3 without 2010 data. Another notable result is that all the five model configurations under-estimated high sediment yields and over-estimated low sediment values w.r.t. the physically based formulation i.e., the GEOtopSed. Although underestimation and overestimation of simulated sediment yields w.r.t. the observation data has been previously reported^37,38^, the following section details the reasons for the expressed trend vis-à-vis simplifications in the process representations. The relative performances of different model configurations are similar when models are calibrated against observed data (Supplementary Fig. S1).

### Physical controls on the differences in SSY estimates

To evaluate the reason for differences in modeled SSY estimates, we study them at the daily scale, the finest temporal resolution for analyses in this study. It is to be noted that SSY estimates at coarser scales are obtained by aggregating daily simulation estimates.

#### Differences in SSY estimates from MUSLE based model configurations

**Figure 2:**
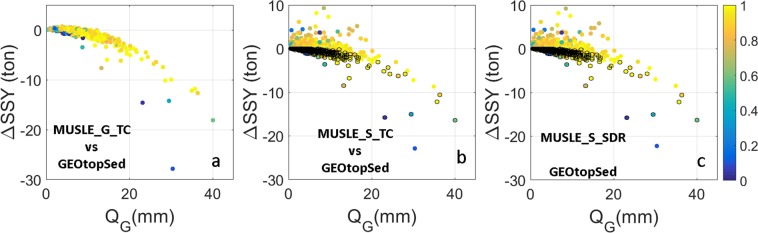
Scatter plots of ΔSSY vs *Q*_*G*_ for different MUSLE based model configurations. ΔSSY represents the difference in SSY estimates between different MUSLE based model configurations and GEOtopSed model. Points are color coded with normalized watershed average antecedent soil moisture ($\hat{\theta }$) in the top 3.5 cm soil layer. Colored dots with black edges indicate underestimation of surface runoff by the MUSLE based model configurations w.r.t. GEOtopSed.

We first take a closer look at the differences in SSY estimates (ΔSSY) between different MUSLE based model configurations and GEOtopSed model (Fig. 2).

**Figure 3:**
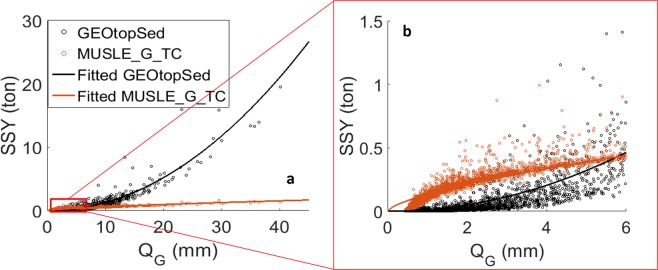
The scatter plot of daily SSY vs. Q_G_ for GEOtopSed and MUSLE_G_TC models (left) and a zoom-in of the left plot for Q_G_ < 6 mm (right).

A negative ΔSSY for large *Q*_*G*_ indicates that MUSLE_G_TC generally underestimates SSY with respect to estimates from GEOtopSed. *Q*_*G*_ is the overland flow discharge estimate from GEOtop model. This can primarily be attributed to the different rates of sediment loss for a given runoff in the two model configurations. Although the MUSLE_G_TC model uses derived statistics of hydrologic states (in form of flow erosivity) from GEOtop model and hence has the same driving force for erosion and deposition calculations as the GEOtopSed, the two models use different erosion and transport capacity calculations (see Eqs (5–10) in Method section). Figure 3 shows the sediment yield from the two model configurations, given identical daily runoff. It is to be noted that the exponent in the power-law term that relates SSY amount and runoff is within the range (from 0.5 to 2.42) reported by Julien and Simons^32^. The MUSLE_G_TC model generated more erosion than GEOtopSed model when the volume of daily discharge (*Q*_*G*_) is less than 6 mm per unit area (Fig. 3b). However, when the flow volume increased beyond this threshold, GEOtopSed model generated more sediment per unit discharge. The differences in the exponent (2.01 for GEOtopSed vs. 0.667 for MUSLE_G_TC) result in significant differences in SSY estimates for larger discharge magnitudes (Fig. 3a). This also explains the reason for a narrower (/broader) range of point spread along the MUSLE_G_TC (/GEOtopSed) axis in Fig. 1. A few blue points at the lower right corner in Fig. 2a indicate that the SSY differences are especially large when the simulated soil moisture is small. This is due to the decrease in soil cohesion with soil saturation^39^, which is evaluated using a localized equation for Dripsey catchment:

4 $${{\rm{\zeta }}}_{s}={(\frac{\theta }{{\theta }_{s}})}^{2}\,{{\rm{\zeta }}}_{ss}$$

in GEOtopSed. Notably, the Eq. (4) in GEOtopSed model is customized to reflect the relation between soil cohesion and soil moisture of the major soil type at Dripsey catchment^12^. The relation may vary with different soil types (e.g. high clay content soil). Here, ζ_*s*_ and ζ_*ss*_ are bare soil cohesion and saturated bare soil cohesion, respectively. *θ* and *θ*_*s*_ are the soil moisture and saturated soil moisture contents. Enhanced erosion in response to large precipitation events on less cohesive dry soil can cause large discrepancies in SSY estimates from MUSLE_G_TC and GEOtopSed, even at annual scale. Since none of the MUSLE based model configurations account for the influence for soil cohesion effects on soil erodibility, they do not capture enhanced erosion due to large precipitation on dry soil. Differences in the characterization of moisture feedback on soil cohesion and the relation between SSY vs. runoff may significantly impact SSY estimates. A detailed example demonstrating soil cohesion impacts on SSY is highlighted in the Supplementary Fig. S5.

**Figure 4:**
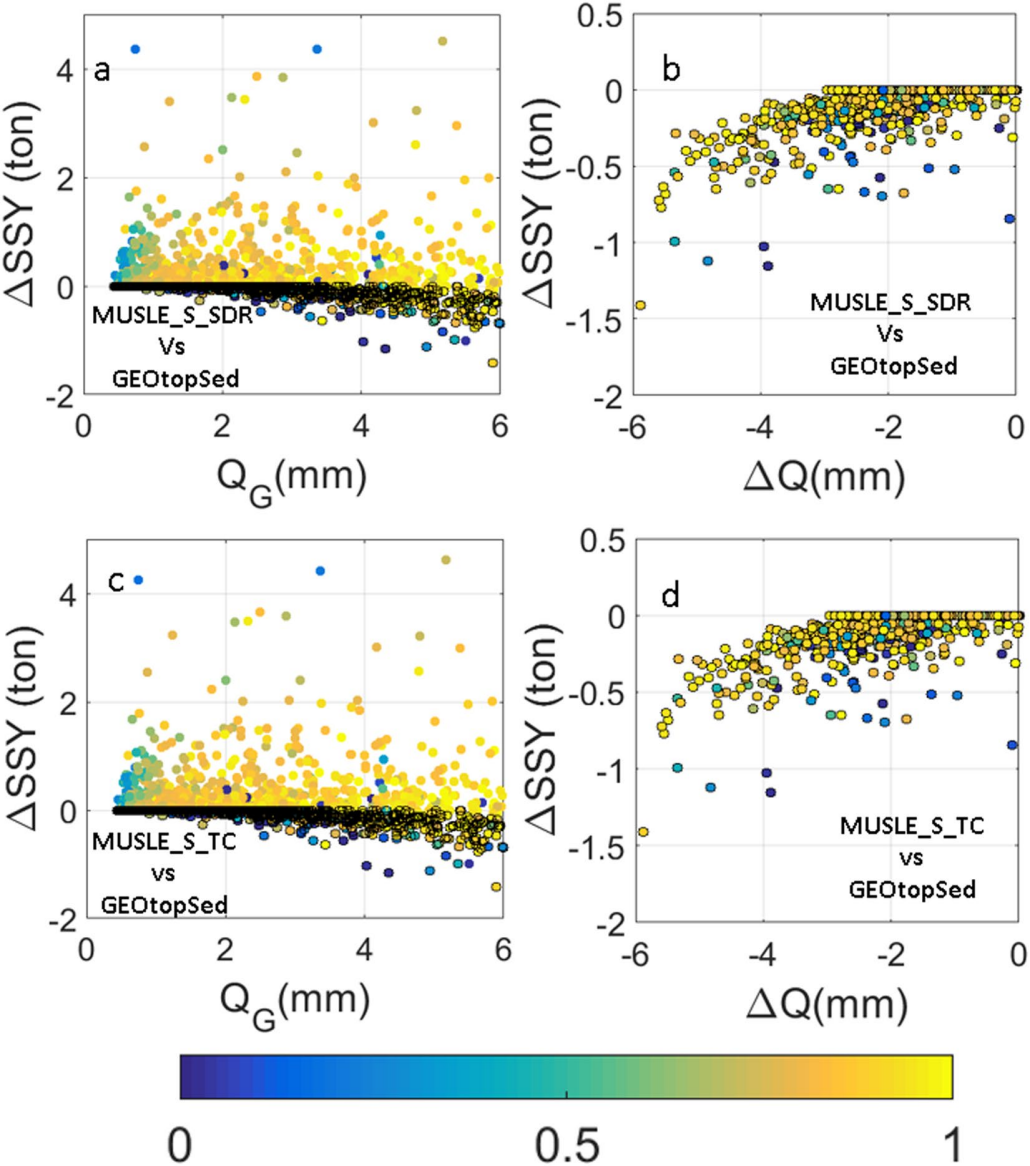
(**a**,**c**) Scatter plots of ΔSSY versus Q_G_ for Q_G_ < 6. (**b**,**d**) Scatter plots of ΔSSY versus ΔQ for Q_G _< 6 and ΔQ < 0. All plots were color coded with normalized watershed average antecedent soil moisture ($\hat{{\boldsymbol{\theta }}}$) in the top 3.5 cm soil layer. Colored dots with black edges indicate underestimation of surface runoff by MUSLE based model configurations w.r.t. GEOtopSed.

For model configurations that use MUSLE and SCS approach for erosion and hydrology calculations respectively (e.g. MUSLE_S_TC and MUSLE_S_SDR), differences in the SSY estimates with respect to GEOtopSed may also arise from the differences in the runoff volume used to drive soil detachment. For example, although the flow simulated by SCS curve number method using the procedure proposed by Sahu, *et al*.^40^ accounts for temporal variations in antecedent conditions, it does not account for the effects of topographic distribution of soil moisture due to lateral groundwater or overland flow. Also, the methodology used previous 5-day cumulative rainfall to account for changes in initial abstraction through time. This empirical scheme produces temporal soil moisture variations that are muted in relation to that obtained by GEOtopSed. Finally, the model does not account for diffusion of runoff peak as it translates over the landscape. These simplifications of hydrological processes in the curve number method result in large discrepancies in generated overland flow (*Q*_*s*_) with respect to the overland flow generated by GEOtopSed (*Q*_*G*_) (*R*^2^ = 0.13, see Supplementary Fig. 7S). In spite of the differences in runoff estimates between GEOtopSed and MUSLE based models, the trend of ΔSSY in Fig. 2b,c look similar to that in Fig. 2a. ΔSSY still shows large negative values for large *Q*_*G*_. Notably, MUSLE_S_TC and MUSLE_S_SDR overestimate SSY for smaller *Q*_*G*_, and the magnitude of positive ΔSSY is much larger than for MUSLE_G_TC configuration. A zoom-in of ΔSSY vs *Q*_*G*_ plot (Fig. 4) shows that ΔSSY is positive when the runoff is overestimated by the MUSLE based model and negative when it is underestimated. Since SSY simulated by MUSLE based model is larger than that by GEOtopSed for identical runoff magnitude and *Q*_*G*_ < 6 mm (see Fig. 3), for days when MUSLE based configurations overestimate runoff, ΔSSY is bound to be positive. If the overland flow is underestimated by MUSLE based method, the decrease in runoff may push ΔSSY to negative value even when *Q*_*G*_ < 6 mm. Figure 4b,d show that for large negative ΔQ, ΔSSY is indeed negative. For negative ΔQ values that are close to zero, ΔSSY might get positive. It is to be noted that some sediment models that use MUSLE and SCS scheme for erosion and hydrology calculations also use a delay factor to route and diffuse runoff peaks^25^. Such models are expected to produce *Q*_*s*_ that has a better correlation with *Q*_*G*_. In that case, one may expect the accuracy of SSY estimates from MUSLE_S_TC model to be better, possibly as high as that from a MUSLE_G_TC model, although MUSLE_S_TC is still likely to be adversely affected by inaccurate soil moisture distribution originating from inadequate representation of the influence of neighboring hydrologic connectivity. The analysis highlights the role of hydrologic process feedback that influences runoff volume, and routing schemes that determine the shape of hydrographs, on SSY estimates. Error in estimation of either the runoff peak or the recession limb of the event hydrograph is likely to result in errors in SSY estimates.

#### Differences in SSY estimates from RUSLE based model configurations

RUSLE based model configurations use derived precipitation statistics in form of rainfall erosivity as a driving force for erosion. Important hydrological states and processes, such as antecedent soil moisture condition, evapotranspiration, infiltration processes, and runoff generation, which are crucial drivers for sediment erosion and transport are not explicitly accounted for in these model configurations. As a result, the correlation between rainfall erosivity, the driver of temporal variations in SSY estimates in RUSLE based models, and overland flow, the driver of SSY variations in GEOtopSed model is poor (*R*^2^ = 0.03, Supplementary Fig. S7). This causes the difference in SSY estimates between RUSLE based models and GEOtopSed to be significant. Notably, the correlation between rainfall erosivity and overland flow, *Q*_*s*_, from SCS method is much better (*R*^2^ = 0.625). This is in part because the SCS scheme used here^40^ to obtain runoff does not account for translation and diffusion of runoff peak and is only moderately impacted by antecedent moisture conditions, which results in *Q*_*s*_ to co-vary strongly with precipitation.

**Figure 5:**
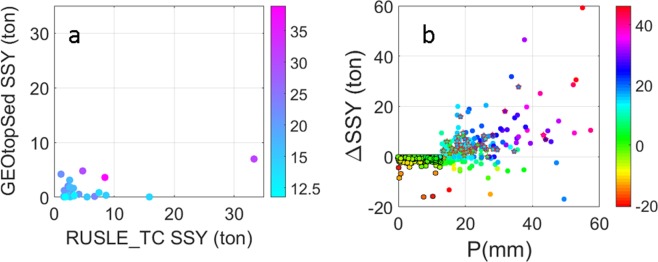
(**a**) Scatter plot of daily SSY by GEOtopSed and RUSLE_TC, color coded with daily precipitation, for events when the spatially-averaged antecedent soil moisture is near saturation ($\hat{{\boldsymbol{\theta }}}$ > 0.95). (**b**) Scatter plot for daily ΔSSY and daily precipitation (P), color coded with (P - Q_G_). Circles with black edge represent P < 12.5 mm. Pentagrams with pink edge are points shown in (**a**).

One source of difference in SSY estimates from the RUSLE model is the definition of erosive rainfall. RUSLE model uses 12.5 mm as a threshold for erosive rainfall, and hence the soil losses during small rainfall events are neglected. Figure 5b shows negative ΔSSY for events with *P* < 12.5 mm, although the magnitude of ΔSSY is small. At near saturation soil moisture conditions ($\hat{\theta }$ > 0.95) i.e. conditions when differences in SSY estimates using RUSLE based and GEOtopSed models is expected to be mostly due to the differences in representation of erosion and deposition processes rather than soil moisture feedback, the RUSLE_TC model generally overestimates SSY with respect to GEOtopSed (Fig. 5a), provided the rainfall (*P*) is larger than the erosive rainfall threshold (12.5 mm) and (*P* − *Q*_*G*_) > 0. This indicates the erosion process representation in the two models is such that for *P* > *Q*_*G*_ and *P* > 12.5 mm, RUSLE based parameterization overpredicts SSY with respect to GEOtopSed. In fact, irrespective of the soil moisture conditions, for *P* > 12.5 mm and large (*P* − *Q*_*G*_), RUSLE_TC overpredicts SSY estimates with respect to GEOtopSed. In contrast, for *P* > 12.5 mm and (*P* − *Q*_*G*_) < 0, ΔSSY is negative (Fig. 5b). It is to be noted that (*P* − *Q*_*G*_) < 0 for *P* > 12.5 mm indicates a situation when precipitation event on the previous day generates a runoff hydrograph (*Q*_*G*_) of intensity much larger than *P* on a given day. This results in estimated SSY from GEOtopSed to be larger than that from RUSLE based model on that given day. The analysis indicates that RUSLE based model configurations are likely to overestimate SSY on the day of large precipitation events but underestimate during runoff recession periods or on the day of small precipitation events (*P* < 12.5 mm), and hence are not well suited for daily simulation of SSY. Also, since these model configurations do not account for the non-linear influence of antecedent moisture conditions and evapotranspiration on runoff generation and soil cohesion, they are unable to capture the temporal variation in SSY, as estimated by GEOtopSed, at monthly to annual scales.

## Conclusions and Synthesis

The study presents an intercomparison experiment to evaluate the role of varied permutations of representation of sediment processes on modeled sediment yield estimates at different temporal scales. The goal was to evaluate the extent to which sediment dynamics as simulated by a physically-based model with coupled surface-subsurface flow interactions, are captured by models with simpler process representations. Six model configurations ranging from a physically-based unit stream-power driven model to an empirical RUSLE equation based model were implemented in a 15 km^2^ maritime catchment at Dripsey, Ireland. Our results show that:

(a) parsimonious and computationally efficient sediment model configuration such as MUSLE_G_TC that uses MUSLE based equation for evaluation of erosion and transport capacity but has an identical hydrological input as the GEOtopSed model (a much more complex physically-based model), can capture more than 60% of variations in SSY at a wide range of temporal scales. If erosion events in response to large daily precipitation on very dry soils are ignored i.e. events when reduced soil cohesion can significantly intensify erosion, Fisher unbiased estimator performance for MUSLE_G_TC models may get larger than 0.8 at monthly to annual scales. This indicates that as long as amplified erosion events in response to intense storms on dry soils are non-existent, a much simpler MUSLE_G_TC model may provide reasonable estimate of SSY at monthly to annual scales as that by computationally intensive GEOtopSed model. On the flip side, MUSLE based models, even when driven using identical hydrological inputs as a physically-based model, may miss more than 35% of the variability in SSY yield time series simulated by the GEOtopSed at daily scale. This is because MUSLE based models underestimate the large SSY events and overestimate small SSY events with respect to GEOtopSed due the differences in the relation used to capture the influence of daily runoff volume and peak flow on sediment yield. Our findings also indicate that there is a potential to significantly improve SSY estimates from MUSLE based models by accounting for the dynamic role of soil cohesion on sediment yield. This would require MUSLE based models to incorporate soil moisture feedback and its impact on soil erodibility, from the coupled hydrologic model;

(b) sediment models with simpler runoff generation representations such as one based on SCS curve number method may not capture the temporal variations of SSY at daily event scale, even when using MUSLE based sediment yield characterizations (e.g. in MUSLE_S_TC or MUSLE_S_SDR), in part because of errors in capturing the event runoff hydrograph. Poor performance of MUSLE_S_TC with respect to MUSLE_G_TC at daily scale indicates that accurate prediction of daily runoff hydrograph can significantly improve SSY estimates. In contrast, the performance is better at coarser (monthly to annual) time scales. This is because at scales longer than an event duration, the performance is mostly determined by the model’s ability to capture the variations in SSY across the entire simulation period. As the SCS curve number method is able to predict larger runoff (and hence larger SSY) during wet periods and smaller runoff during dry periods at monthly and annual scales, the model performance is better at these scales even when appreciable errors exist at daily scales;

(c) there is no obvious improvement in the model performance depending on if the SSY estimates were obtained using an explicit sediment routing method such as MUSLE_S_TC vs. a SDR based method such as MUSLE_S_SDR. This indicates that if the goal is to predict SSY, between MUSLE_S_TC and MUSLE_S_SDR configurations, either may be used without significant loss of accuracy. Similarity in the performances of these two model configurations is aided by the homogeneity of the catchment and the forcing. Heterogeneous distribution of LULC, on the other hand, would cause the power-law relation between SDR and topographic properties to vary over the landscape. This in turn will result in differences in MUSLE_S_TC and MUSLE_S_SDR simulations, as the transport capacity calculation in MUSLE_S_TC model configuration (see Eq. (10)) does not explicitly account for this heterogeneity.

It is worth pointing out that transport capacity based representation allows identification of the net sediment gain/loss locations while SDR based representation only returns soil loss. However, MUSLE_S_SDR is expected to be much more computationally efficient than MUSLE_S_TC as it does not require calculation of transport capacity and explicit evaluation of deposition on every grid;

(d) between model configurations using RUSLE and MUSLE schemes, MUSLE based models such as MUSLE_S_SDR and MUSLE_S_TC have significantly better correlations with GEOtopSed model than RUSLE based schemes at all considered temporal scales. This is largely because RUSLE based equation tends to overestimate the impact of large precipitation events on SSY. Also, since RUSLE based model do not account for the diffusivity of runoff hydrograph and the moderating impact of temporally evolving antecedent moisture conditions and evapotranspiration on runoff peak, they end up overestimating SSY yield from large events. Since, SSY estimates in RUSLE equation are only evaluated for precipitation events that are larger than 12.5 mm per day, these models underestimate SSY yields for smaller events. Notably, the discrepancies at daily scale may get large enough to make RUSLE based equation ill-suited to even simulate inter-annual temporal variations in SSY.

Comparisons with observation data showed similar relative performance of different model configurations as evidenced through model intercomparisons, and hence did not affect our conclusions (Supplementary Fig. S1). Aforementioned conclusions were derived based on model application in Dripsey catchment. The runoff generation at Dripsey catchment is mostly due to saturation-excess runoff mechanism, which in turn is strongly influenced by the soil moisture feedback. Hence, the performances of some MUSLE/RUSLE model configurations that do not adequately account for soil moisture feedback, yield poor results. These model configurations are expected to perform better in settings where extreme precipitation events that generally lead to infiltration-excess runoff are the dominant contributor to sediment yield. Performances are also likely to be better in settings with low soil surface permeability. Alarmingly, over time, models based on MUSLE/RUSLE formulations (see examples in Tables 3 and S2) have been indiscriminately used, without consideration of the dominant runoff mechanism of the study area.

**Table 3:** Selected model configurations and corresponding process representations.

Model configurations	Detachment	Deposition	Transport
GEOtopSed	GEOtop (3)	TC (C3)	ii
MUSLE_G_TC	GEOtop (2)	TC (C2)	ii
MUSLE_S_TC	SCS (2)	TC (C2)	ii
RUSLE_TC	EI30 (1)	TC (C1)	ii
MUSLE_S_SDR	SCS (2)	SDR (B)	i
RUSLE_SDR	EI30 (1)	SDR (B)	i

Representation type (see Table 1 for more details) is shown in parenthesis.

The selected model configurations used in this study only address the land surface soil loss and transport processes. The processes such as soil particles transport in streams, bank erosion, clustering and resuspension were not considered. While the homogeneity of the selected catchment facilitated interpretation of results, on the flip side it prevented study of the influence of heterogeneous land cover and forcings on the derived conclusions. To test the translatability of the analyses and conclusions presented here, future studies should focus on implementing the considered model configurations in a range of hydroclimatic settings. It is to be noted that although the considered model configurations encompass a broad range of process representations (see Tables 1 and S2), given that there can be many other alternative formulations for each representation type, not all representations can be and has been covered in our analyses. For example, the “Type C3” representation of deposition process (see Table 1) can be formulated using Eq. (6) based on DeRoo, *et al*.^8^ or according to Eq. (1)^41^. Future studies may extend the intercomparison experiments further by incorporating other commonly used process representations.

In spite of aforementioned limitations, the study facilitates understanding of the limitations introduced by process representations in sediment models and can be used to guide selection of appropriate sediment models at different temporal scales. The results may also be used to help prioritize model improvement efforts, as the study clearly identifies processes and states that strongly influence sediment yield estimates and highlights ways to improve simpler sediment models without introducing the complexities inherent in detailed physically-based models. Furthermore, the results may help in prioritization of resources for field campaigns and observation systems for constraining model uncertainties. For example, distributed measurements of soil moisture and its influence on soil cohesion, which play a crucial role in determining runoff response, can be used in assimilation, for improved prediction of sediment yield.

## Method

### Selected model configuration and process representations

Considering that multiple sediment yield models (details in Supplementary Table S2) use some combination of the process representations listed in Table 1, a total of six sets of process representations (hereafter referred as model configurations) are assessed (Table 3). Starting from a physically-based coupled sediment and hydrology model configuration, rest five configurations use increasingly simplified representation of runoff generation, detachment, deposition and transport processes. As the simplification is implemented in one or few processes at a time, this design for intercomparison of model configurations is expected to highlight the role of simplified process representations on modeled sediment yield. For example, as GEOtopSed and MUSLE_G_TC (see Table 3) use identical hydrologic states but different representations for erosion and transport capacity, comparison between these two model configurations is expected to highlight the influence of representation of erosion and transport capacity processes on suspended sediment yield (SSY) estimates. Similarly, the comparison between MUSLE_G_TC and MUSLE_S_TC, which differ only in their characterization of runoff processes, is expected to demonstrate the role of runoff generation mechanism on estimates of sediment yield. Next, we present the details of the six model configurations and highlight benefits of specific intercomparisons.

The first model configuration (Table 3) is an integrated physically-based model of hydrology and sediment transport called GEOtopSed^12^. GEOtopSed uses GEOtop model framework^42,43^ to simulate spatially-distributed soil moisture in each subsurface layer by solving the variably saturated 3D Richards equation. After updating the soil moisture profile in each time step, the head gradient in the top soil layer is used for estimation of infiltration/exfiltration flux. Then surface overland flow is routed based on the elevation gradient. Similar process characterizations, though with varied representations of coupling between surface and subsurface processes, have been used in several state of the art hydrologic models^44–46^ to model processes and states in a range of settings^47^. Using forcings and overland flow variables from GEOtop, GEOtopSed explicitly solves for rainfall splash detachment (*D*_*r*_), flow detachment (*D*_*f*_) and deposition (*D*_*P*_) using the strategy detailed in DeRoo, *et al*.^8^. A similar configuration has been used in some other physically-based coupled models of hydrology and sediment dynamics such as tRIBs-OFM^10^ and InHM^11^. *D*_*f*_ and *D*_*P*_ are related to transport capacity (*TC*) based on the erosion-deposition theory proposed by:

5 $${D}_{f}=y{v}_{s}(TC-C)$$

6 $${D}_{P}={v}_{s}(TC-C)$$

where *y* is an efficiency coefficient that is a function of soil cohesion^8^, and *v*_*s*_ is settling velocity of the particles. The transport capacity is based on the experiments conducted by Govers^22^:

7 $$TC=a\,{(\omega -{\omega }_{cr})}^{b}\rho $$

where *ω* is the unit stream power [m s^−1^]^48^, *ω*_*cr*_ is the critical power that initiates flow detachment of soil particles [m s^−1^], *ρ* is the density of soil particles [kg m^−3^], and *a* and *b* are empirical parameters related to soil particle size. Readers interested in knowing more about process characterizations in GEOtopSed should refer to Zi, *et al*.^12^.

The next model configuration (Table 3), MUSLE_G_TC, uses hydrologic states predicted by GEOtopSed but instead of performing explicit calculations of flow and splash detachment as in Type 3 detachment process and Type C3 deposition process (see Table 1 for more details), erosion is quantified using a Modified Universal Soil Loss Equation (MUSLE) based scheme^20^ i.e. using Type 2 detachment process and Type C2 deposition representation. Here, total erosion or detachment (*D*_*E*_) is calculated using method from MUSLE:

8 $${D}_{E}=\sum _{i}\,{F}_{{e}_{i}}K\,LS\,{C}_{f}\,{P}_{f}$$

where *F*_*ei*_ is flow erosivity at the ith grid, *K* is soil erodibility, *LS* is length- slope factor, *C*_*f*_ is land cover-management factor and *P*_*f*_ is support practice factor. The flow erosivity is obtained using:

9 $${F}_{{e}_{i}}={({Q}_{i}{q}_{pea{k}_{i}})}^{0.56}$$

Runoff volume (*Q*) and peak flow (*q*_*peak*_) in MUSLE_G_TC is derived based on runoff estimates from GEOtopSed. The transport capacity is obtained using^30^:

10 $$TC=ktc\,{F}_{{e}_{i}}\,K\,D{A}^{1.4}\,{S}^{1.4}$$

where *ktc* is a calibration factor, *DA* is drainage area, and S is slope. As GEOtopSed and MUSLE_G_TC use identical hydrologic states but different process representations for erosion and transport capacity (explicit splash and flow detachment calculations vs. flow erosivity based calculations), comparison of SSY estimates from these two model configurations is expected to highlight the influence of representations of erosion and transport capacity processes.

The third model configuration is MUSLE_S_TC. It is similar to MUSLE_G_TC in every way with the only difference being that the flow variables in MUSLE_S_TC are obtained using SCS curve number based method, instead of from GEOtop model. Cumulative runoff volume (*Q*_*a*_) using SCS curve number method^21^ is obtained using:

11 $${Q}_{a}=\frac{{({P}_{a}-{I}_{a})}^{2}}{P-{I}_{a}+Sr}$$

12 $$Sr=25.4(\frac{1000}{CN}-10)$$

where *I*_*a*_ is the initial abstraction, *P*_*a*_ is the cumulative precipitation, *Sr* is the potential maximum retention, and *CN* is the curve number. The curve number is used to account for the effects of land cover and antecedent soil moisture on runoff generation. The peak flow rate (*q*_*peak*_) is generally derived using rational formula:

13 $${q}_{peak}=\frac{{a}_{tc}\,Q\,DA}{3.6\,{t}_{conc}}$$

where *DA* is drainage area, *Q* is the daily runoff volume, and *a*_*tc*_ is the fraction of daily rainfall that occurs during the time of concentration (*t*_*conc*_). MUSLE_S_TC configuration has been used in models such as WASA-SED^49^ and LandSoil^50^. The comparison between MUSLE_G_TC and MUSLE_S_TC is expected to demonstrate the role of runoff characterization on estimates of sediment yield.

Process representations are further simplified in the fourth model configuration, RUSLE_TC. Herein, *E* and *TC* is calculated using exactly the same equations (Eqs (8) and (10)) as used in MUSLE_G_TC and MUSLE_S_TC. But instead of using flow erosivity (*F*_*ei*_), rainfall erosivity (*R*_*ei*_) is used. *R*_*ei*_ in RUSLE_TC is obtained by:

14 $${R}_{{e}_{i}}=EI{30}_{i}$$

where *EI*30 is a compound erosivity index of total storm kinetic energy and maximum 30 minutes rainfall intensity that is calculated on daily basis. Models that use RUSLE_TC configuration include STREAM^51^ and WATEM/SEDEM^31^. Comparison of RUSLE_TC with either MUSLE_G_TC or MUSLE_S_TC is expected to highlight the influence on simulated sediment dynamics by erosion estimation method, i.e., if it is done based on rainfall erosivity vs. flow erosivity.

Two additional model configurations, viz., MUSLE_S_SDR and RUSLE_SDR, are considered. These two model configurations use same equations for evaluating erosion as is used in MUSLE_S_TC and RUSLE_TC, respectively. However, MUSLE_S_SDR and RUSLE_SDR do not explicitly account for deposition and sediment transport. Instead, a SDR multiplication factor that approximates the average portion of eroded soil that is transported out of the area of interest is used to evaluate SSY. The SDR is calculated using the equation proposed by Williams^52^:

15 $$SDR=1.355\times {10}^{-11}\,{k}_{SDR}\,D{A}^{-0.0998}\,r{l}^{0.3629}\,C{N}^{5.444}$$

where *k*_*SDR*_ is a calibration factor, *rl* is the relief and *CN* is the curve number. Models that use RUSLE_SDR and/or MUSLE_S_SDR configurations include SEDD^53^. Comparisons between MUSLE_S_TC and MUSLE_S_SDR or between RUSLE_TC and RUSLE_SDR will highlight the role of transport capacity based deposition scheme vs. one based on SDR on sediment yield estimates.

All six model configurations simulate soil loss in a spatially explicit fashion.

### Model intercomparison strategy

The six considered model configurations are intercompared at a small experimental catchment, at Dripsey, Ireland. GEOtopSed is the most data intensive of the six model configurations. Data needs for other model configurations is just a subset of the input data for GEOtopSed. More details about the site and the data used to populate these models are presented in the SI sections titled “Site description” and “Model data”. Evaluation of the influence of process representations on sediment yield involved intercomparison of SSY estimates at the catchment outlet from GEOtopSed to that from the other five model configurations. SSY at the catchment outlet from GEOtopSed and model configurations with name ending with “TC” (e.g., MUSLE_G_TC, MUSLE_S_TC and RUSLE_TC) are a result of sediment routing mediated by flow transport capacity. SSY from model configurations with name ending with “SDR” (e.g., MUSLE_S_SDR and RUSLE_SDR) are summation of the product of soil loss and sediment delivery ratio at each grid in the catchment. Identical grid resolution is used across the six configurations to facilitate intercomparison without the confounding effects of variable grid resolution. The considered experiment design is suitable for addressing the primary goal of this study, which is to evaluate the influence of simpler process representations on sediment yield estimates. It is to be noted here that the study does not use comparison of modeled SSY to the observation data for analyses. This is because of two main reasons: (a) the comparison of simulation results to observation data is influenced by the uncertainty in the hydrogeological and climatological data used for modeling^54^, their representation on the model grid^55–57^, and calibration of parameters. Since the amount of data used by different process representations is different, so will be the uncertainty introduced because of them. Similarly, even if an identical automated calibration tool is used for different model configurations, its effectiveness for identifying optimal parameters varies with model complexity^58,59^; (b) the daily suspended sediment concentration data in the selected catchment exists only for the period ranging from Jan 1st, 2002 to Dec 31st, 2003. The two-year data period precludes comparison of the simulated SSY from different model configurations with observation data at coarser temporal scales (e.g. seasonal and annual), as the sample data for comparison will be very small. These factors are likely to interfere with the interpretation of how process representations of erosion, deposition and transport processes affect sediment yield estimates. However, for reference, scatter plots showing observed and modeled SSY estimates at daily and monthly scales are presented in the Supplementary Fig. S1. Six model configurations were calibrated against observed data to have similar SSY yield.

Given that GEOtopSed incorporates the most detailed process characterization of hydrology and sediment processes among the considered model configurations, and has been calibrated and validated at both plot and catchment scales^12^, here we ran GEOtopSed for an eleven year period (2002 to 2012) to obtain multiyear estimates of SSY. The validation results of GEOtopSed model can be found in Supplementary Fig. S4. Long term SSY estimate from GEOtopSed was then used as a base data against which estimates from all other model configurations were compared.

SSY estimates from GEOtopSed were compared to that from the other five model configurations at daily, monthly, seasonal and annual scale (Fig. 1). Fisher approximate unbiased estimator^60^ was used to evaluate the correlation between models to account for the different sample sizes across temporal scales:

16 $$F=r+\frac{1-{r}^{2}}{2N}r.$$

where *F* is Fisher estimator, *r* is correlation coefficient, and *N* is the sample size. Fisher estimator closer to 1 indicates a higher degree of agreement in the SSY estimates.

## Supplementary information

Supplementary Information

## Data Availability

The model code and datasets generated during and/or analyzed during the current study are available from the corresponding author on request.
