# Supplementary material for: Intercomparing varied erosion, deposition and transport process representations for simulating sediment yield
Source: Sci Rep. 2019 Aug 19;9:12029. doi: 10.1038/s41598-019-48405-9 (PMC6700289; doi:10.1038/s41598-019-48405-9)
Supplement: Supplementary file 1 — Supplementary Information [file 41598_2019_48405_MOESM1_ESM.docx]

**Intercomparing varied erosion, deposition and transport process representations for simulating sediment yield**

Tan Zi^1^, Mukesh Kumar^2^*, and John Albertson^3^

1. Tetra Tech Inc, Integrated Water Management, Fairfax, 22030, USA
2. University of Alabama, Civil, Construction and Environmental Engineering, Tuscaloosa, 35487, USA
3. Cornell University, Civil and Environmental Engineering, Ithaca, 14853, USA

*Corresponding author

E-mail: mkumar4@eng.ua.edu

**Supplementary Information**

**Symbols list**

*The following symbols are used in this paper:*

*P* Rainfall

$EI_{30}$ $E$ is rainfall energy and $I_{30}$is maximum 30 minutes rainfall intensity

*Q* Flow volume

*q_peak_* Peak flow rate

$R_{e}$ Rainfall erosivity

$F_{e}$ Flow erosivity

SCS Soil Conservation Service

$D_{r}$ Rain drop detachment

$D_{f}$ Flow detachment

$D_{P}$ Deposition

$D_{E}$ Total detachment

$TC$ Transport capacity

$C_{S}$ Sediment concentration

$E$ Rainfall energy

USLE Universal Soil Loss Equation

RUSLE Revised Universal Soil Loss Equation

SDR Sediment delivery ratio

SSY Suspended sediment yield

MUSLE_G_TC Model configuration using MUSLE, GEOtop hydrological output and Transport Capacity calculations

MUSLE_S_TC Model configuration using MUSLE, SCS, and Transport Capacity calculations

MUSLE_S_SDR Model configuration using MUSLE, SCS, and SDR calculations

RUSLE_TC Model configuration using RUSLE and Transport Capacity calculations

RUSLE_SDR Model configuration using RUSLE and SDR calculations

$Q_{G}$ Overland flow discharge estimate from GEOtop model

$Q_{s}$ Overland flow discharge estimate from SCS method

$\theta$ Soil moisture

$\theta_{s}$ Saturated soil moisture

$\hat{\theta}$ Normalized watershed average antecedent soil moisture

${}_{s}$ Bare soil cohesion

${}_{ss}$ Saturated bare soil cohesion

$y$ Efficiency coefficient that is a function of soil cohesion

$v_{s}$ Settling velocity of the particles

$\omega$ Unit stream power

$\omega_{cr}$ Critical power that initiates flow detachment of soil particles

$\rho$ The density of soil particles

$a$ Empirical parameter related to soil particle size

$b$ Empirical parameter related to soil particle size

$K$ Soil erodibility

$LS$ Length- slope factor

$C_{f}$ Land cover-management factor

$P_{f}$ Support practice factor

$ktc$ Calibration factor for transport capacity

$DA$ Drainage area

$S$ Slope

$Q_{a}$ Cumulative runoff volume

$I_{a}$ Initial abstraction

$P_{a}$ Cumulative precipitation

$Sr$ Potential maximum retention

$CN$ Curve number

$t_{conc}$ Time of concentration

$a_{tc}$ Fraction of daily rainfall that occurs during the time of concentration

*DOY* Day of year

$k_{SDR}$ Calibration factor for SDR

$rl$ The relief

$F$ Fisher estimator

$r$ Correlation coefficient

$N$ Sample size

**Site description**

The Dripsey catchment is located approximately 25 km northeast of Cork and has an area of 15 km^2^ (Fig. S6). The elevation of this catchment ranges from 60 to 210 m. It is a beef and dairy producing agricultural catchment and is almost 100% covered by perennial ryegrass. The catchment slopes gently, with around 85% of the area having less than 3% grade. Gleys and podzols are the two major soil types. The climate in the study region is temperate maritime and is characterized by high humidity and a lack of temperature extremes during the year. The minimum daily temperature for years 2002 and 2003 was -0.2°C. The mean annual precipitation locally is approximately 1400 mm. Data from 2002 to 2012 show that on an average, October to January receives more rainfall than other months Fig.S3. It shows the probability of daily precipitation. 75% of daily rainfall was less than 6.52mm and 95% was less than 18.4mm. Only 13 days during the 11 years have precipitation larger than 40mm, 5 of them have precipitation larger than 50mm.

**Model data**

The models require a digital elevation model (DEM), land use/land cover (LULC) map, and soil type map to simulate the hydrological processes for a catchment. The soil type map and soil parameters were obtained from Irish Forestry soils (IFS) database and in situ soil samples. LULC parameters were derived based on classifications in the Corine land cover 2000 database and land use data observed in the catchment. The stream channel was delineated using DEM processing in GIS. The derived extent of stream was validated against the regional channel map. Geomorphic properties of the channel were defined based on the DEM data. All thematic maps were resampled at 50 m × 50 m, the spatial resolution of the models. Meteorological data such as precipitation, temperature, incoming shortwave radiation, air pressure, relative humidity, wind speed and direction in half-hourly time steps were collected at the flux tower in Dripsey [^1^](#_ENREF_1). Because of the small size of the catchment (area = 15km^2^) and absence of any other precipitation data fine enough to resolve the heterogeneities within the catchment, the rainfall was assumed to be uniform within the catchment. The assumption is reasonable given the mild topographic relief and a uniform land cover within the catchment. Overall, the catchment is homogeneous in terms of parameters and forcing, and serves as an ideal test case to intercompare model configurations because of its simple setup. At the catchment outlet (elevation 60 m), stream flow was monitored continuously at 30 minutes interval for a period of over two years (2002-2003). Hourly sediment concentrations were derived from the flow-weighted water samples at the catchment outlet that were collected for the two year period using an ISCO 6712 auto-sampler with intake set at approximately 0.25 m above the streambed. Daily streamflow and sediment concentration data sets were used for calibration and validation of the GEOtopSed in the catchment [^2^](#_ENREF_2).

For other five model configurations, precipitation records were used to derive rainfall erosivity. Flow erosivity was calculated using equations (9), (11)-(13). The LULC, soil type and texture information were used to estimate parameters such as $K$, $C_{f}$, and $P_{f}$. $K$ factor was estimated using the equation proposed by [Williams, et al. ^3^](#_ENREF_3):

|  | $K=0.2+0.3 \exp\left( 0.0256 Sa \left( 1-\frac{Si}{100} \right) \right) \left( \frac{Si}{Cl+Si} \right)^{0.3} \left( 1-0.25 \frac{OC}{OC+\exp\left( 3.72-2.95 OC \right)} \right) (1-0.7 \frac{SN}{SN+\exp\left( -5.51+22.9 SN \right)})$ | (S1) |
| --- | --- | --- |

where $Sa$, $Si$, $Cl$, and $OC$ are percentages of sand, silt, clay and, organic carbon. $SN$ is the non-sand percentage of soil. These soil fractions were obtained from the soil samples. The product of $C$ and $P$ factor was set equal to 0.09 for pasture, as used in [Panagos, et al. ^4^](#_ENREF_4). DEM, and slope data were used to evaluate $LS$, $DA$, and $rl$. Three parameters, $ktc$ , $CN$ , and $k_{SDR}$, which are used in equations (10), (12) and (15) respectively were obtained through calibration. Calibration of parameters were performed using a linear search scheme such that the eleven year streamflow and SSY estimate from each of the five RUSLE/MUSLE based model configurations were as close as possible to that obtained from the GEOtopSed model.

**Example of soil cohesion impacts on erosion**

The overestimation of SSY in 2010 by GEOtopSed w.r.t. other considered model configurations (Fig. 1) is largely due the overestimation of erosion during a 10 days period (day of year 10 to 19) in early winter. In this period, the catchment received around 112 mm of rainfall, with maximum daily precipitation of 49.4 mm (6th largest daily rainfall during the entire simulation period). In the GEOtopSed model, the rainfall events generated 72.92% of SSY for the year 2010 (Fig. S5a). In contrast, the SSY portion for RUSLE_TC, MUSLE_S_TC and MUSLE_G_TC were 11.55%, 22.41%, and 14.28% respectively. The reason for extremely large SSY yield by GEOtopSed during the 10 days period is the low antecedent soil moisture conditions. Dry antecedent soil conditions led to smaller soil cohesion (see equation (4)) at the beginning of 10 days period (Fig. S5b), thus increasing soil erodibility. This is especially true when the soil cohesion is less than 15 Kpa (Fig. S5c). See [Zi, et al. ^2^](#_ENREF_2) for detailed sensitivity analyses of SSY vis-à-vis soil cohesion.

**Table S1 Fisher unbiased estimator of all model configurations versus observed data at monthly and daily scales.**

| **Model Comparisons** | **Monthly** | **Daily** |
| --- | --- | --- |
| RUSLE_SDR vs Obs | 0.4081 | 0.2543 |
| MUSLE_S_SDR vs Obs | 0.7724 | 0.4794 |
| RUSLE_TC vs Obs | 0.4081 | 0.2543 |
| MUSLE_S_TC vs Obs | 0.7727 | 0.4798 |
| MUSLE_G_TC vs Obs | 0.9301 | 0.7552 |
| GEOtopSed vs Obs | 0.9532 | 0.8466 |

**Table S2: A list of sediment models and schemes used for representing detachment, deposition and transport processes within them. See Table 1 for details about representation types.**

| **Model** | **Representation type for detachment processes** | **Representation type for deposition processes** | **Representation type for transport processes** | **Temporal resolution applied in listed references** | **Reference** |
| --- | --- | --- | --- | --- | --- |
| AGNPS | 1 | C2 | ii | Event | [^5^](#_ENREF_5) |
| ANSWERS | 2 | C2 | ii | Event | [^6^](#_ENREF_6) |
| CASC2D-SED | 2 | C2 | ii | Event | [^7^](#_ENREF_7) |
| CREAMS | 1 and 2 | C3 | ii | Multi-years | [^8^](#_ENREF_8) |
| EROSION 2D/3D | 3 | C3 | ii | Sub-event | [^9^](#_ENREF_9) |
| EUROSEM | 3 | C3 | ii | Sub-event | [^10^](#_ENREF_10) |
| GEOtopSed | 3 | C3 | ii | Sub-event | [^11^](#_ENREF_11) |
| GUEST | 3 | C3 | ii | Sub-event | [^12^](#_ENREF_12)) |
| HSPF | 2 | B | ii | Sub-event | [^13^](#_ENREF_13) |
| InHM | 3 | C3 | ii | Sub-event | [^14^](#_ENREF_14) |
| KINEROS | 3 | C3 | ii | Sub-event | [^15^](#_ENREF_15) |
| KINEROS2 | 3 | C3 | ii | Sub-event | [^16^](#_ENREF_16) |
| LandSoil | 2 | C2 | ii | Multi-years | [^17^](#_ENREF_17) |
| LASCAM | 2 | C2 | ii | Daily | [^18^](#_ENREF_18) |
| LISEM | 3 | C3 | ii | Sub-event | [^19^](#_ENREF_19) |
| MHYDAS-Erosion | 3 | C3 | ii | Sub-event | [^20^](#_ENREF_20) |
| MUSLE | 2 | A | i | Daily | [^21^](#_ENREF_21) |
| PERFECT | 2 | A | i | Daily | [^22^](#_ENREF_22) |
| RUSLE | 1 | A | i | Multi-years | [^23^](#_ENREF_23) |
| RUSLE2 | 1 | C2 | ii | Long term daily average | [^24^](#_ENREF_24) |
| SEDD | 1 | B | ii | Multi-years | [^25^](#_ENREF_25) |
| SHESED | 3 | C3 | ii | Sub-event | [^26^](#_ENREF_26) |
| SWAT | 2 | A | ii | Daily | [^27^](#_ENREF_27) |
| SWRRB | 1 and 2 | C3 | ii | Daily | [^28^](#_ENREF_28) |
| tRIBS-OFM | 3 | C3 | ii | Sub-event | [^29^](#_ENREF_29) |
| USLE | 1 | A | i | Multi-years | [^30^](#_ENREF_30)^,^[^31^](#_ENREF_31) |
| WASA-SED | 1 | C2 | ii | Multi-years | [^32^](#_ENREF_32) |
| WATEM/SEDEM | 1 | C1 | ii | Multi-years | [^33^](#_ENREF_33)^,^[^34^](#_ENREF_34) |
| WEPP | 3 | C3 | ii | Sub-event | [^35^](#_ENREF_35) |

*
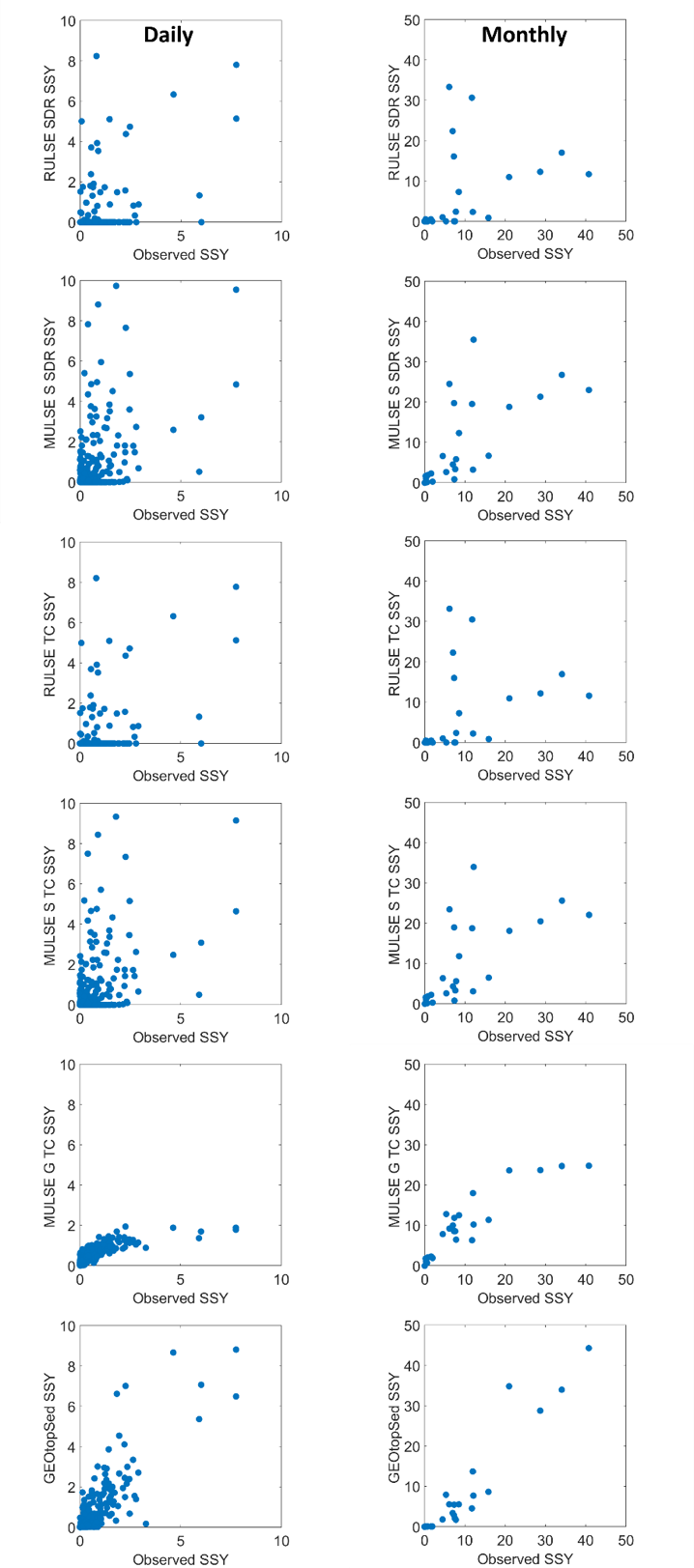
*

**Figure S1: Scatter plots comparing observed and modeled SSY estimates (ton/time interval) for all six model configurations at daily (left column) and monthly (right column) scales. Fisher unbiased estimator for these comparisons are shown in Table S1.**


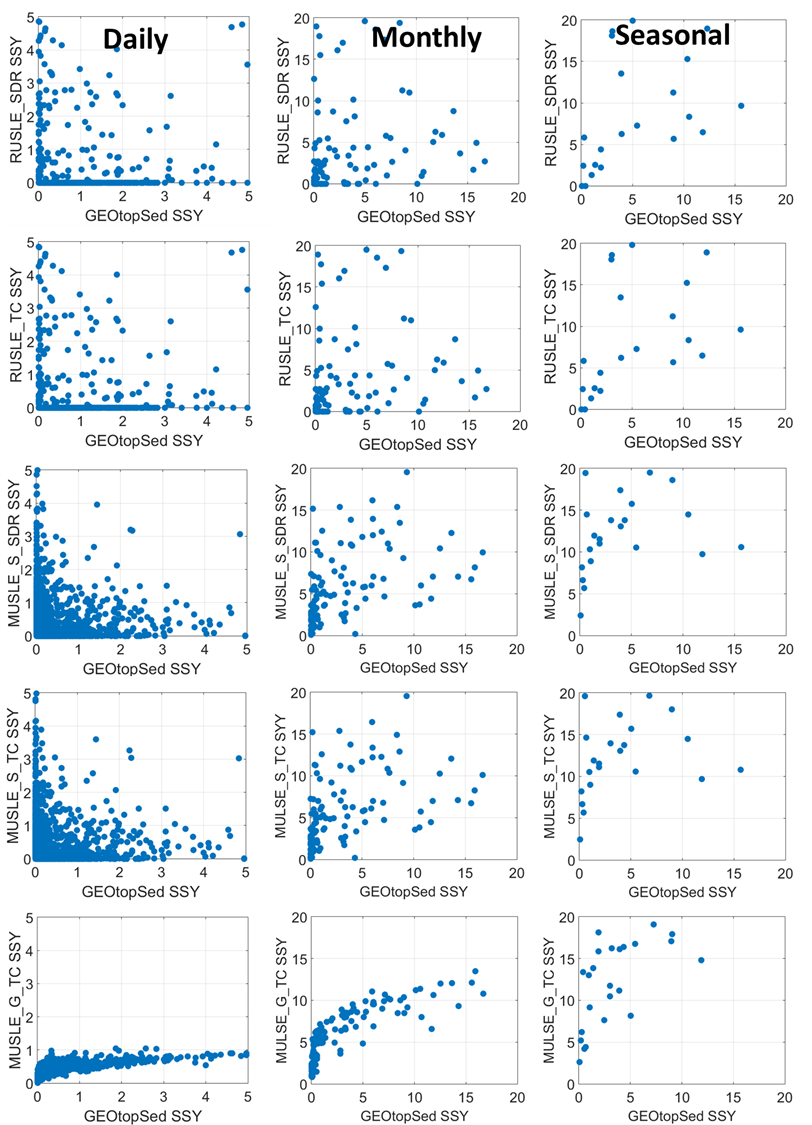


**Figure S2: Zoom-in scatter plots comparing SSY estimates (ton/time interval) between GEOtopSed and five other model configurations at daily, monthly, and seasonal scales.**


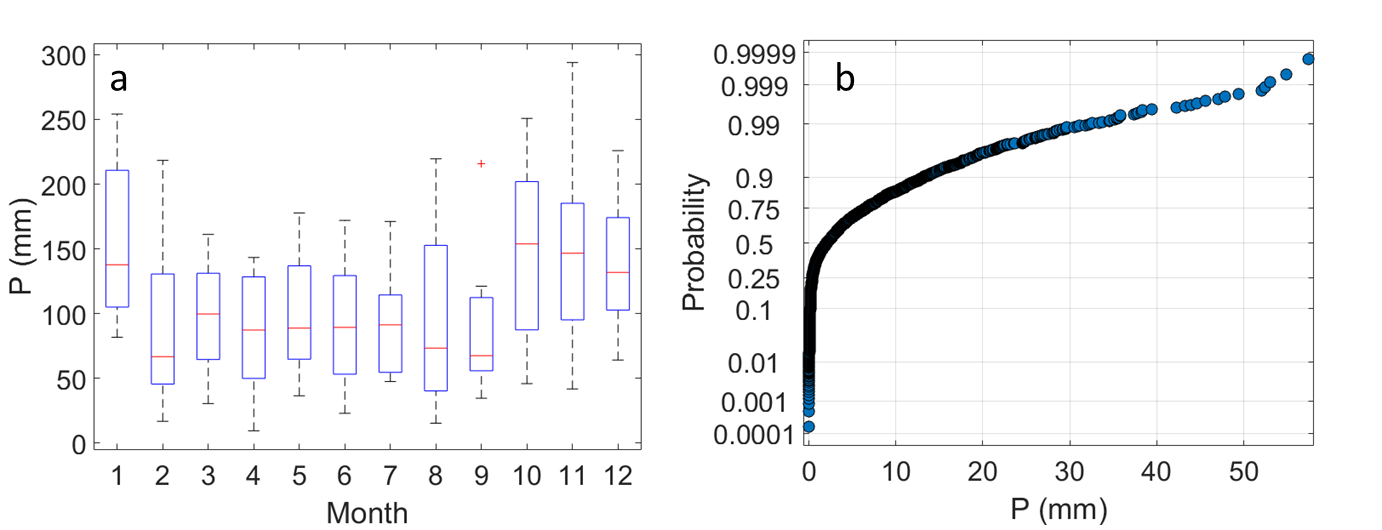


**Figure S3: (a) Multi-year (2002-2012) monthly average rainfall and (b) Daily rainfall probability plot for Dripsey catchment**


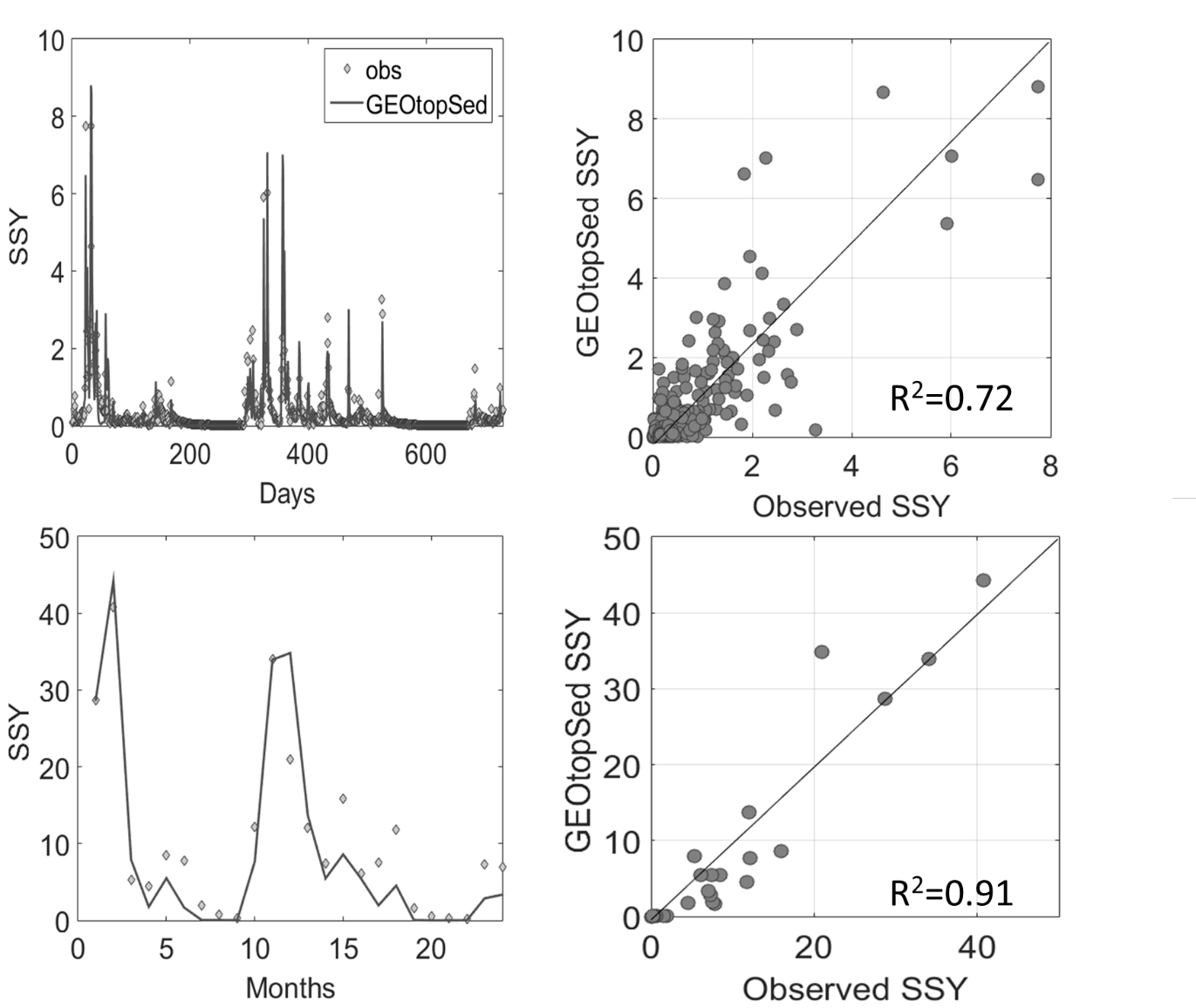


**Figure S4: Comparison of daily and monthly GEOtopSed results and observation**


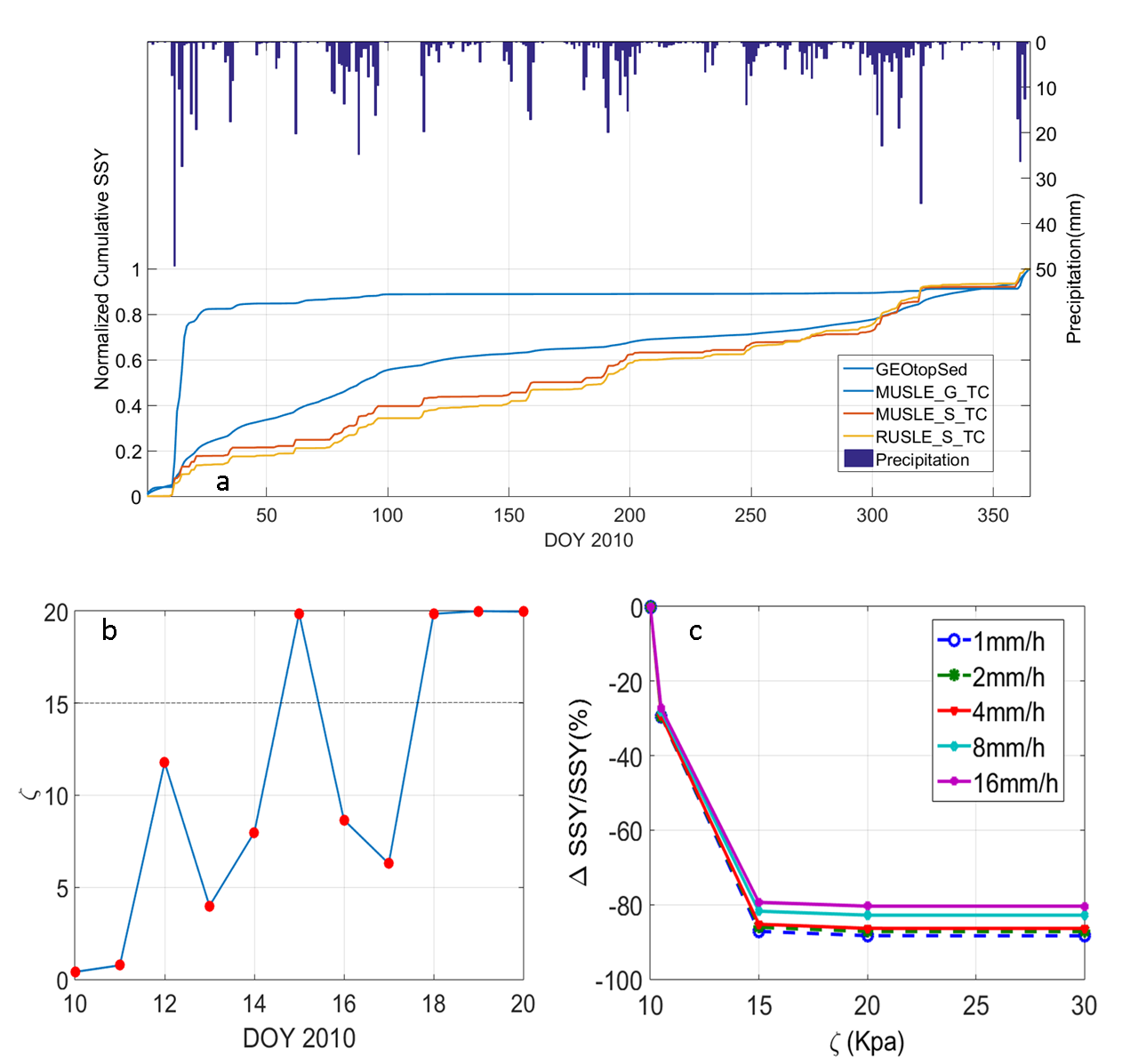


**Figure S5: (a) Normalized cumulative SSY for four model configurations. The cumulative SSY plot for MUSLE_S_TC and RUSLE_S_TC completely overlap that of MUSLE_S_SDR and RUSLE_S_SDR respectively, and hence they have not been drawn here; (b) Variation of soil cohesion during the 10 wet days (from Jan. 10 to Jan. 20) in 2010; and (c) Sensitivity analyses of SSY w.r.t. soil cohesion.**


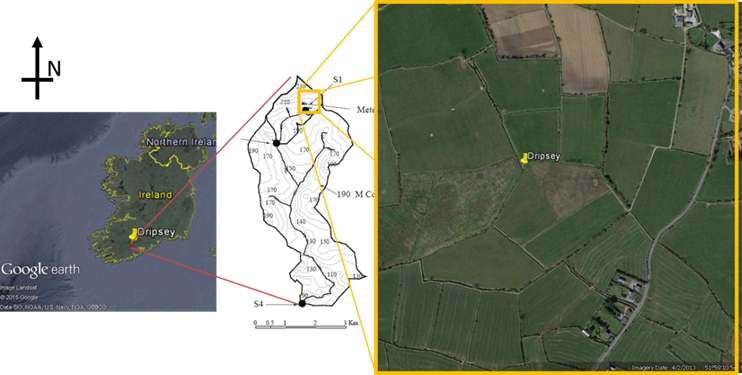


**Figure S6: Location of Dripsey catchment in County Cork, Ireland. The yellow square is the location of flux tower（Map data: Google, Image Landsat, DigitalGlobe）.**


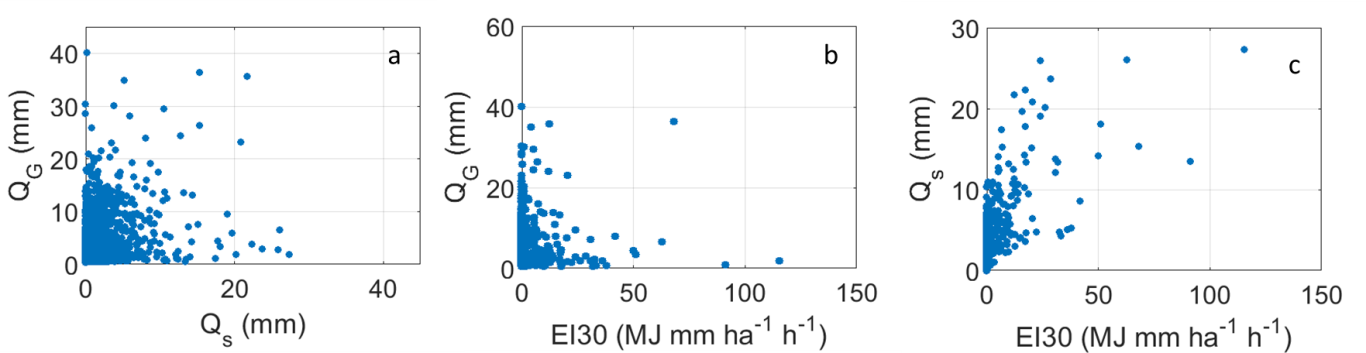


**R^2^=0.625**

**R^2^=0.03**

**R^2^=0.13**

**Figure S7: Scatter plot of (a) daily overland flow simulated by GEOtopSed (**$\boldsymbol{Q}_{\boldsymbol{G}}$**) and curve number method (**$\boldsymbol{Q}_{\boldsymbol{s}}$**); (b) daily overland flow simulated by GEOtopSed (**$\boldsymbol{Q}_{\boldsymbol{G}}$**) and rainfall erosivity (EI30); (c) daily overland flow simulated by curve number method (**$\boldsymbol{Q}_{\boldsymbol{s}}$**) and rainfall erosivity (EI30).**

**Reference**

1 Albertson, J. D. & Kiely, G. On the structure of soil moisture time series in the context of land surface models. Journal of Hydrology 243, 101-119, doi:10.1016/s0022-1694(00)00405-4 (2001).

2 Zi, T., Kumar, M., Kiely, G., Lewis, C. & Albertson, J. Simulating the spatio-temporal dynamics of soil erosion, deposition, and yield using a coupled sediment dynamics and 3D distributed hydrologic model. Environmental Modelling & Software 83, 310-325, doi:https://doi.org/10.1016/j.envsoft.2016.06.004 (2016).

3 Williams, J. R., Renard, K. G. & Dyke, P. T. EPIC: A new method for assessing erosion's effect on soil productivity. Journal of Soil and water Conservation 38, 381-383 0022-4561 (1983).

4 Panagos, P. et al. Estimating the soil erosion cover-management factor at the European scale. Land Use Policy 48, 38-50 0264-8377 (2015).

5 Young, R. A., Onstad, C. A., Bosch, D. D. & Anderson, W. P. AGNPS: A nonpoint-source pollution model for evaluating agricultural watersheds. J. Soil Water Conserv. 44, 168-173 (1989).

6 Beasley, D. B., Huggins, L. F. & Monke, a. ANSWERS: A model for watershed planning. Transactions of the ASAE 23, 938-0944 (1980).

7 Johnson, B. E., Julien, P. Y., Molnar, D. K. & Watson, C. C. The two‐dimensional upland erosion model casc2d‐sed1. JAWRA Journal of the American Water Resources Association 36, 31-42 (2000).

8 Knisel, W. G. CREAMS : a field scale model for chemicals, runoff and erosion from agricultural management systems. (U.S.D.A., 1980).

9 Schmidt, J. A mathematical model to simulate rainfall erosion. Catena Suppl 19, 101-109 (1991).

10 Morgan, R. P. C. et al. The European Soil Erosion Model (EUROSEM): A dynamic approach for predicting sediment transport from fields and small catchments. Earth Surface Processes and Landforms 23, 527-544 (1998).

11 Zi, T., Kumar, M., Kiely, G., Lewis, C. & Albertson, J. Simulating the spatio-temporal dynamics of soil erosion, deposition, and yield using a coupled sediment dynamics and 3D distributed hydrologic model. Environmental Modelling & Software in review (2016).

12 Rose, C., Coughlan, K. & Fentie, B. in Modelling Soil Erosion by Water Vol. 55 NATO ASI Series (eds John Boardman & David Favis-Mortlock) Ch. 30, 399-412 (Springer Berlin Heidelberg, 1998).

13 Donigian Jr, A., Bicknell, B., Imhoff, J. & Singh, V. Hydrological Simulation Program-Fortran (HSPF). Computer models of watershed hydrology., 395-442 (1995).

14 Heppner, C. S., Ran, Q., VanderKwaak, J. E. & Loague, K. Adding sediment transport to the integrated hydrology model (InHM): Development and testing. Adv. Water Resour. 29, 930-943, doi:10.1016/j.advwatres.2005.08.003 (2006).

15 Smith, R. E., Goodrich, D. C., Woolhiser, D. A., Unkrich, C. L. & Singh, V. P. KINEROS-A kinematic runoff and erosion model. Computer models of watershed hydrology., 697-732 (1995).

16 Goodrich, D. C., Unkrich, C. L., Smith, R. E. & Woolhiser, D. A. in Hydrologic modeling for the 21st Century agenda: Second Federal Interagency Hydrologic Modeling Conference, July 28-August 1, 2002, Riviera Hotel, Las Vegas, Nevada. ([Reston, Va.: United States Interagency Advisory Committee on Water Data] the Subcommittee on Hydrology, 2002.).

17 Ciampalini, R., Follain, S. & Le Bissonnais, Y. LandSoil: A model for analysing the impact of erosion on agricultural landscape evolution. Geomorphology 175–176, 25-37, doi:<http://dx.doi.org/10.1016/j.geomorph.2012.06.014> (2012).

18 Viney, N. R. & Sivapalan, M. A conceptual model of sediment transport: application to the Avon River Basin in Western Australia. Hydrological Processes 13, 727-743, doi:10.1002/(SICI)1099-1085(19990415)13:5<727::AID-HYP776>3.0.CO;2-D (1999).

19 DeRoo, A. P. J., Wesseling, C. G. & Ritsema, C. J. LISEM: A single-event physically based hydrological and soil erosion model for drainage basins .1. Theory, input and output. Hydrological Processes 10, 1107-1117 (1996).

20 Gumiere, S. J. et al. MHYDAS-Erosion: a distributed single-storm water erosion model for agricultural catchments. Hydrological Processes 25, 1717-1728, doi:10.1002/hyp.7931 (2011).

21 Williams, J. R. in Present and Prospective Technology for Predicting Sediment Yield and Sources Vol. ARS-S-40 244-252 (U.S. Dept. Agric., 1975).

22 Littleboy, M. et al. Impact of soil erosion on production in cropping systems .I. Development and validation of a simulation model. Soil Research 30, 757-774, doi:<http://dx.doi.org/10.1071/SR9920757> (1992).

23 Renard, K. G., Foster, G. R., Weesies, G. A. & Porter, J. P. RUSLE: revised universal soil loss equation. Journal of soil and Water Conservation 46, 30-33 0022-4561 (1991).

24 Foster, G. et al. Revised Universal Soil Loss Equation Version 2. Science Documentation.(Draft). USDA-ARS (2005).

25 Ferro, V., Porto, P. Sediment Delivery Distributed (SEDD) Model. Journal of Hydrologic Engineering 5, 411-422, doi:doi:10.1061/(ASCE)1084-0699(2000)5:4(411) (2000).

26 Wicks, J. M. & Bathurst, J. C. SHESED: a physically based, distributed erosion and sediment yield component for the SHE hydrological modelling system. Journal of Hydrology 175, 213-238, doi:<http://dx.doi.org/10.1016/S0022-1694(96)80012-6> (1996).

27 Arnold, J. G., Srinivasan, R., Muttiah, R. S. & Williams, J. R. Large area hydrologic modeling and assessment part i: Model development. JAWRA Journal of the American Water Resources Association 34, 73-89, doi:10.1111/j.1752-1688.1998.tb05961.x (1998).

28 Arnold, J. G., Williams, J., Nicks, A. & Sammons, N. SWRRB; a basin scale simulation model for soil and water resources management. (Texas A & M University Press, 1990).

29 Kim, J., Ivanov, V. Y. & Katopodes, N. D. Modeling erosion and sedimentation coupled with hydrological and overland flow processes at the watershed scale. Water Resour. Res. 49, 5134-5154, doi:10.1002/wrcr.20373 (2013).

30 Wischmeier, W. H. A Rainfall Erosion Index for a Universal Soil-Loss Equation1. Soil Science Society of America Journal 23, doi:10.2136/sssaj1959.03615995002300030027x (1959).

31 Wischmeier, W. H. & Smith, D. D. Predicting rainfall erosion losses-A guide to conservation planning. Predicting rainfall erosion losses-A guide to conservation planning (1978).

32 Medeiros, P. H., Güntner, A., Francke, T., Mamede, G. L. & Carlos de Araújo, J. Modelling spatio-temporal patterns of sediment yield and connectivity in a semi-arid catchment with the WASA-SED model. Hydrological Sciences Journal–Journal des Sciences Hydrologiques 55, 636-648 (2010).

33 Van Oost, K., Govers, G. & Desmet, P. Evaluating the effects of changes in landscape structure on soil erosion by water and tillage. Landscape Ecology 15, 577-589, doi:10.1023/A:1008198215674 (2000).

34 Van Rompaey, A., Verstraeten, G., Van Oost, K., Govers, G. & Poesen, J. Modelling mean annual sediment yield using a distributed approach. Earth Surface Processes and Landforms 26, 1221-1236 (2001).

35 Laflen, J. M., Lane, L. J. & Foster, G. R. WEPP: A new generation of erosion prediction technology. J. Soil Water Conserv. 46, 34-38 (1991).
